# Parent perceptions of their child’s and their own physical activity after treatment for childhood cancer

**DOI:** 10.1007/s00520-022-07288-9

**Published:** 2022-08-01

**Authors:** Lauren Ha, Claire E. Wakefield, Joanna Fardell, Richard J. Cohn, David Simar, Christina Signorelli, David Mizrahi

**Affiliations:** 1 School of Health Sciences, UNSW Medicine and Health, UNSW Sydney, Sydney, Australia; 2 Kids Cancer Centre, Sydney Children’s Hospital, Randwick, Australia; 3 School of Clinical Medicine, UNSW Medicine and Health, UNSW Sydney, Sydney, Australia; 4 Prince of Wales Clinical School, UNSW Medicine and Health, UNSW Sydney, Sydney, Australia; 5 The Daffodil Centre, The University of Sydney, A Joint Venture With Cancer Council NSW, Sydney, Australia

**Keywords:** Parent perceptions, Child, Childhood cancer, Physical activity

## Abstract

**Purpose:**

Parents are important facilitators of physical activity for children, yet little is known about the perceptions of parents of childhood cancer survivors. We investigated parent perceptions of their own and their child’s physical activity levels after cancer treatment and examined associations with clinical, demographic, and psychosocial factors.

**Methods:**

We conducted a cross-sectional survey among 125 parents and 125 survivors. Parents reported on the perceived importance of their child being physically active and concerns regarding exercising after cancer treatment.

**Results:**

Parents and survivors self-reported median (range) of 127.5 (0–1260) and 220 (0–1470) min/week of moderate-to-vigorous physical activity. Most parents (*n* = 109, 98%) believed that physical activity was highly important for their child. Some parents (*n* = 19, 17%) reported concerns, most commonly regarding exercise safety (*n* = 7, 22%). Parents were more likely to perceive that their child should increase physical activity if their child was an adolescent and had high body fat percentage.

**Conclusions:**

Physical activity levels varied widely among survivors, reflecting factors including parents’ lifestyles, limited understanding of exercise benefits and perceptions of risk. Given survivors’ insufficient physical activity levels and sedentary behaviour among families, embedding physical activity promotion into health systems and follow-up support could benefit the entire family unit.

## Introduction

The survival rate for childhood cancer is approximately 80% [1]. Despite recent advances in cancer therapies, many childhood cancer survivors are faced with substantial risks of treatment-related late effects [2]. Obesity, cardiovascular disease, type II diabetes mellitus, and poor quality of life are common late effects experienced by survivors of childhood cancer [3, 4]. These late effects can be exacerbated by insufficient physical activity levels and reduced cardiorespiratory fitness, commonly observed among survivors [5, 6]. Limited recreational or structured physical activity in this population may result in a more sedentary lifestyle, thus increasing survivors’ risk for developing comorbid lifestyle diseases, e.g. heart disease, osteoporosis, and cancer reoccurrence. Given the increased risk of comorbidities and the global challenge of achieving sufficient physical activity levels, promoting regular physical activity among young people is a priority, particularly for cancer survivors [7, 8].

Understanding social and environmental factors that influence physical activity supports development of effective programs to increase physical activity levels in children and adolescents [9]. Parents can be important facilitators for improving health behaviours in children [9]. Previous studies in the general population have investigated the influence of parents on their children’s participation in physical activity and suggest that parents play an influential role on their child’s behaviours [10–12]. Parents can offer resources to engage in physical activity, provide positive reinforcement for participation, and play a role in eliminating barriers to physical activity for their children [11]. Social support, such as having a physical activity role model, has also been shown to positively influence physical activity levels among adult cancer patients and survivors [13]. Given this positive impact, parents may influence physical activity behaviour in childhood cancer survivors.

The influence of parents has also been investigated in previous research through examining the relationship between parents’ moderate-to-vigorous physical activity (MVPA) and their child’s MVPA. Some studies have found strong correlations between parent and child MVPA [14, 15], while other studies found no relationship [16–18]. The link between parent MVPA and their child’s MVPA may be influenced by other factors; however, this association has not yet been explored within the childhood cancer population.

Another potential factor of parent influence on children’s physical activity levels is how parents perceive the value of physical activity for their child. Previous research indicates that parents’ perceptions play a significant role in their child’s physical activity levels [16, 19]. One study demonstrated that parents’ perceptions about their child’s physical competence significantly influenced their child’s MVPA [16]. The psychosocial functioning of parents may also influence their perceptions of physical activity for their child. In the general population, the consequences of parental distress and depression can lead to unsupportive parenting practices [20], less time spent with children [21], and reduced social support [22]. These parenting behaviours were found to be associated with unhealthy behaviours and greater weight gain in children [23, 24]. Parent overprotection has also been linked to poorer outcomes in children diagnosed with cancer, such as child distress and reduced health-related quality of life [25, 26]. Given the level of psychological distress parents of children with cancer experience during and after treatment, [27] it is possible that parent functioning may similarly influence perceived importance and engagement with physical activity of childhood cancer survivors.

Therefore, our aims were to (1) identify parent perceptions of their own physical activity levels and investigate any association between parents’ and survivors’ self-reported physical activity levels, (2) investigate any association between parents’ psychosocial functioning and survivors’ self-reported physical activity levels, (3) investigate how parents perceive their child’s physical activity levels, including the perceived importance of physical activity, parents’ desire to increase physical activity for their child, and any concerns regarding exercising after cancer treatment, and (4) identify clinical and psychosocial factors associated with parent perceptions that their child should increase their physical activity levels. For aim 1, we hypothesised that higher levels of parent self-reported physical activity would be associated with higher levels of survivors’ physical activity. For aim 2, we hypothesised that parents’ positive psychosocial outcomes would be associated with higher levels of survivors’ self-reported physical activity. For aim 3, we hypothesised that parents value the importance of physical activity for their child yet may have concerns for their child exercising after cancer treatment. For aim 4, we hypothesised that parents’ perceptions about their child’s physical activity levels would be influenced by parents’ psychosocial functioning and their child’s clinical factors including age, diagnosis, treatment exposure, time since treatment completion, and adiposity.

## Methods

### Study design

We conducted a cross-sectional study to explore parent perceptions toward their child’s physical activity levels after completion of cancer treatment. The data from this study were collected as a part of a larger study, evaluating the accuracy of childhood cancer survivors’ perceptions on their own physical activity and fitness levels [6].

### Participants

We recruited parents from the Sydney Children’s Hospital, Australia between September 2017 and March 2020 (i.e. prior to the COVID-19 pandemic significantly affecting daily life in Australia). One parent per family was eligible to participate if they could read English, and their child was 18 years old or younger at study enrolment, had been diagnosed with any type of cancer and had completed cancer treatment at least 12 months prior. Nursing staff and the lead researcher identified potential participants from oncology clinic lists. Final eligibility was confirmed by the treating consultant. Parents of eligible participants were contacted by telephone prior to their child’s routine clinic visit and provided informed written consent on the day of their clinic appointment, before commencing any study procedures. Parents who opted into the study completed paper surveys during their child’s routine clinic visit. This study was approved by the Sydney Children’s Hospital Network Human Research Ethics Committee (LNR/16/SCHN/403 and HREC/18/SCHN/471).

### Data collection

We used a previously published survey that included questions on parent demographic information including age, sex, income, educational attainment, and postcode [28]. We asked parents to report on their child’s demographic and clinical information including age, sex, cancer diagnosis, and cancer treatment(s) received.

We asked parents and survivors to report on their own physical activity levels using the Godin-Shephard Leisure-Time Physical Activity Questionnaire, a four item questionnaire on time spent in light, moderate, and vigorous physical activity during a typical week [29]. We revised the questionnaire to include minutes to calculate minutes per week for each intensity so that we could compare to the recommended physical activity guidelines. The leisure time activity score corresponds to physical activity metabolic equivalents (METs) and was calculated by multiplying and summing the frequencies of each intensity of activity (9 × strenuous + 5 × moderate + 3 × mild) [29]. The Godin-Shephard leisure-time physical activity questionnaire has been used extensively in oncology research [30] and is a reliable and valid self-report tool for children and adolescents, with test–retest reliabilities of *r* = 0.81 [31]. Responses were categorised into three groups with definitions and examples: ‘strenuous exercise’ (i.e. heart beats rapidly and unable to speak while exercising, e.g. running, jogging, hockey, football), ‘moderate exercise’ (i.e. not exhausting, and still able to talk while exercising, e.g. fast walking, baseball, tennis), and ‘mild exercise’ (i.e. minimal effort, easy to talk while exercising, e.g. yoga, archery, fishing). Body composition including body fat percentage was measured in survivors using bioelectrical impedance (InBody 570) and body mass index (BMI) was calculated based on weight measured on weight scales and height measured using a stadiometer in survivors.

To assess parent psychosocial factors, we used the Parent and Family Adjustment Scale (PAFAS) [32]. The PAFAS is a 30-item parent-report survey measuring parenting practices and parental adjustment. For the purpose of this study, we only used questions from the parental adjustment domain (‘I feel stressed or worried’, ‘I feel happy’, ‘I feel sad or depressed’, ‘I feel satisfied with my life’, and ‘I cope with the emotional demands of being a parent’). Each item is scored on a 4-point scale from ‘not true of me at all’ (0) to ‘true of me very much’ (3), with higher scores indicating worse parental adjustment. The items, ‘I feel happy’, ‘I feel satisfied with my life’, and ‘I cope with the emotional demands of being a parent’ must be reverse scored (i.e. 0 = 3, 1 = 2, 2 = 1, 3 = 0). The PAFAS has displayed good internal consistency alpha of 0.7 to 0.87 across all subscales [32].

We assessed parent perceptions toward their child’s physical activity levels using questions that were previously used in this population [28]. We assessed parents’ perceptions of their child’s current physical activity levels, whether their child should increase their physical activity levels, the importance of their child being physically active and whether they had any concerns for their child exercising after cancer treatment. Questions included parent perceptions toward their child’s physical activity levels (‘How do you feel about the amount of physical activity your child is doing?’) with three response options (doing the right amount, should be doing more, should be doing less); and parents’ perceptions toward their child increasing their physical activity levels (‘Would you like your child to do more exercise?’) using a 4-point Likert scale (not at all, somewhat, probably, definitely). We used a rating scale (0–100) to assess the importance that parents placed on their child being physically active, (‘How important is it to you that your child is physically active?’) with 0 = not important and 100 = extremely important [28]*.* Finally, we assessed whether parents had any concerns about their child exercising after finishing cancer treatment using a short open-ended response (‘Do you have any concerns for your child exercising after cancer treatment?’).

### Data analysis

We analysed data using IBM SPSS Statistics version 26.0 (Armonk, NY: IBM Corp). We compared parents’ and survivors’ self-reported MVPA to the recommended physical activity guidelines for adults over 18 years of age (≥ 150 min/week MVPA) and for children under 18 years of age (≥ 60 min/day MVPA or ≥ 420 min/week MVPA) [33]. We classified participants’ rurality using the Accessibility/Remoteness Index of Australia, which categorises postcode regions according to their accessibility of services [34]. We grouped regions into ‘major city’, ‘inner regional’, and ‘outer regional’.

We used Kendall’s tau to determine aim (1) the relationship between self-reported parent and self-reported survivor MVPA and aim (2) the relationship between parent psychosocial functioning (PAFAS parental adjustment subscale) and self-reported survivor MVPA. We calculated the PAFAS scores to determine parental psychosocial functioning, with higher scores indicating worse parental adjustment. For parent perceptions toward their child’s physical activity levels, we examined the data using descriptive statistics including frequencies, interquartile range (IQR) and percentages of responses. For parent perceptions of their child’s physical activity levels, we dichotomised the outcomes into ‘doing the right amount’ versus ‘should do more’ physical activity. We excluded the outcome, ‘should do less’ physical activity due to the small sample (*n* = 4). We used logistic regression to identify clinical and demographic factors associated with parents perceiving that their child ‘should do more’ physical activity (versus ‘doing the right amount’ of physical activity). The predictor variables were parents’ psychosocial outcomes using scores from the PAFAS subscale, and survivor attributes (age at study, months since treatment completion, cancer treatment, and body fat percentage). We fit univariable models for each predictor individually, as well as two separate multivariable models that included (i) all of the psychosocial outcomes and (ii) all of the survivor attributes. We categorised survivors’ age at study into ‘child’, defined as 8 to 12 years and ‘adolescent’, defined as 13 to 18 years due to differences in parent influence between children and adolescents [35]. ‘Liquid’ tumours were defined as cancers involving the blood, blood-forming organs such as the bone marrow, and lymph nodes. ‘Solid’ tumours were defined as tumours involving the organs other than the haematopoietic system. *P* values < 0.05 were considered statistically significant.

Two researchers (LH and DM) independently reviewed all open-ended responses about parent concerns relating to physical activity for commonly arising themes and categories. We used content analysis to identify the number of concerns in commonly presented themes [36]. Any disagreements between coders were resolved through discussion. As concerns were collated from an open-ended question, not all parents spontaneously addressed every theme.

## Results

We approached 176 eligible participants, of whom 125 parent–child dyads participated (71% recruitment rate). Fourteen participants declined (*n* = 7 child not interested, *n* = 3 parent not interested, *n* = 2 distance barrier, *n* = 1 too busy, and *n* = 1 child with cardiac limitations), and 36 were unreachable. Most parents were female (79%) and from metropolitan areas (83%). Survivors were 40% female (*n* = 50). The most common diagnosis among survivors was acute lymphoblastic leukaemia (*n* = 58, 46%). Nearly all survivors received chemotherapy (*n* = 123, 98%), followed by surgery (*n* = 89, 71%). Table 1 presents demographic and clinical data on both parents and survivors.

**Table 1:** Participant demographic and clinical characteristics

Characteristics	Parents (*n* = 125)
Parent attributes	
Age (years), *mean* (*SD*)^a^	45.4 (5.6)
Sex (female), *n* (*%*)	99 (79.2)
Highest education, *n* (*%*)^b^	
High school	23 (19.0)
Certificate or diploma	44 (36.4)
University degree	54 (44.6)
Rurality, *n* (*%*)^c^	
Metropolitan	104 (83.2)
Inner regional	15 (12.0)
Outer regional	6 (4.8)
Meeting MVPA guidelines, *n* (*%*)^d^	
Sedentary (0 min/week)	29 (23.6)
Below recommendations (1–149 min/week)	37 (30.1)
Meeting recommendations (150 + min/week)	57 (46.3)
Parent-reported MVPA (min/week), *median* (*range*)	127.5 (0–1260)
Godin-Shephard leisure-time physical activity LSI^e^, *median* (*range*)	
Mild activity	6 (0–21)
Moderate activity	10 (0–35)
Strenuous activity	0 (0–63)
Total score	25 (0–119)
Parental adjustment subscale (PAFAS), *n* (*%*)^f^	
Stress or worry	92 (83.0)
Happy	112 (100.0)
Sad or depressed	62 (56.0)
Satisfied with life	111 (99.0)
Coping	108 (98.0)
Child and disease attributes	
Survivor age at study completion (years), *mean* (*SD*)	12.9 (3.3)
Survivor sex (female), *n* (*%*)	50 (40.0)
Survivor diagnosis, *n* (*%*)	
Acute lymphoblastic leukaemia	58 (46.4)
Other malignancies^g^	18 (14.4)
Hodgkin’s lymphoma	13 (10.4)
Neuroblastoma	11 (8.8)
Wilms’ tumour	9 (7.2)
Acute myeloid leukaemia	6 (4.8)
Brain cancer	3 (2.4)
Hepatoblastoma	3 (2.4)
Rhabdomyosarcoma	3 (2.4)
Non-Hodgkin lymphoma	1 (0.8)
Treatments received, *n* (*%*)^h^	
Chemotherapy	123 (98.4)
Radiotherapy	33 (26.2)
Surgery	89 (70.6)
Bone marrow transplant	27 (21.4)
Godin-Shephard leisure-time physical activity LSI^e^, *median* (*range*)	
Mild activity	15 (0–21)
Moderate activity	15 (0–35)
Strenuous activity	18 (0–63)
Total score	46.5 (0–119)
Child MVPA recommendations, *n* (*%*)^*i*^	
Sedentary (0 min/week)	3 (2.6)
Below recommendations (1–419 min/week)	87 (75.7)
Meeting recommendations (420 + min/week)	25 (21.7)
Self-reported MVPA (min/week), *median* (*range*)	220 (0–1470)
BMI (kg/m^2^), *mean* (*SD*)	21.0 (5.0)
Body fat percentage, *mean* (*SD*)	21.7 (10.8)

*BMI*, body mass index; *LSI*, leisure score index; *MVPA*, moderate to vigorous PA; *N*, number of participants; *PAFAS*, Parenting and Family Adjustment Scale. ^a^Missing data for 5 parents’ ages. ^b^Missing data for 4 parents’ education attainment. ^c^We classified participants’ rurality using the Accessibility/Remoteness Index of Australia, which categorises regions according to their accessibility of services. We grouped regions into ‘major city’, ‘inner regional’, and ‘outer regional’ [34]. ^d^Missing data for one parent MVPA. Levels of MVPA were compared against recommended PA guidelines for adults aged 18 or older (at least 150 min/week of MVPA). ^e^Missing data for one parent and 10 survivors’ self-reported physical activity data. LSI interpretation: active (24 + units), moderately active (14–23 units), and insufficiently active/sedentary (< 14 units) [29]. ^f^Missing data for 13 parent scores for the PAFAS. Each item is scored on a 4-point scale from ‘not true of me at all’ (0) to ‘very much true of me’ (3). Proportions represent parents who scored 1–3 on the scale. ^g^Other malignancies include germ cell tumour (*n* = 1), acute lymphoblastic lymphoma (*n* = 1), chronic myeloid leukaemia (*n* = 1), and soft tissue sarcoma (*n* = 1). ^h^Some survivors may have received more than one treatment. ^i^Missing data for 10 survivors’ MVPA. Levels of MVPA were compared against recommended PA guidelines for children and adolescents aged 8–17 years (at least 420 min/week of MVPA). We compared MVPA against adult PA guidelines (at least 150 min/week of MVPA) for survivors aged 18 years old

### Reported physical activity levels in parents and survivors

Parents self-reported that they achieved a mean (*SD*) of 179.1 (207.0) min/week of MVPA (Table 1). Most parents (54%) were not meeting the recommended physical activity guidelines: 24% (*n* = 29) reported being sedentary (0 min/week) and 30% (*n* = 37) were below the recommendations (1–149 min/week), while 46% (*n* = 57) were meeting the recommendations (≥ 150 min/week).

Survivors self-reported that they achieved a mean (*SD*) of 294.1 (260.8) min/week of MVPA. Most survivors (*n* = 90, 78%) were not meeting physical activity guidelines of at least 420 min/week of MVPA for survivors aged 8–17 years old and at least 150 min/week of MVPA for survivors aged 18 years old.

Regarding our first aim, we observed a positive weak correlation between parents’ self-reported MVPA and survivors’ self-reported MVPA (*n* = 115, *Τ* = 0.17, *P* = 0.01).

### Parent psychosocial functioning

Regarding our second aim, we did not observe a significant correlation between parents’ psychosocial functioning and survivors’ self-reported MVPA (*n* = 104, *Τ* =  − 0.10, *P* = 0.16).

### Parent perceptions toward their child’s physical activity levels

For our third aim, parents placed a high level of importance for their child to be physically active (mean score 94/100 where 100 = extremely important, *IQR* 90–100, 83% of parents scored between 90 and 100, Table 2).

**Table 2:** Parent perceptions regarding their child’s physical activity

Survey questions and categories of responses	Parents (*n* = 125)
“How important is it to you that your child is physically active?”^a^	Mean (IQR)
(*0* = *not important*, *100* = *extremely important*)	94/100 (90–100)
Responses	*n*** (%)**
50–69	2 (1.8)
70–89	17 (15.3)
90–100	92 (82.9)
“How do you feel about the amount of PA your child is doing now?”^b^	
They should be doing less	4 (3.3)
They do the right amount	56 (45.5)
They should be doing more	63 (51.2)
“Would you like your child to be doing more PA?”^c^	
Definitely	54 (45.4)
Probably	23 (19.3)
Somewhat	27 (22.7)
Not at all	15 (12.6)
“Do you have any concerns for your child exercising after cancer treatment?”^d^, number of ‘yes’ responses	19 (17)

^a^Missing 14 parent data. ^b^Missing 2 parent data. ^c^Missing 6 parent data. ^d^Missing 13 parent data

Many parents reported that their child ‘should do more’ physical activity (*n* = 63, 51%), while nearly half (*n* = 56, 46%) perceived their child was ‘doing the right amount’ (Table 2). Few parents reported that their child ‘should be doing less’ physical activity (*n* = 4, 3%).

**Fig. 1:**
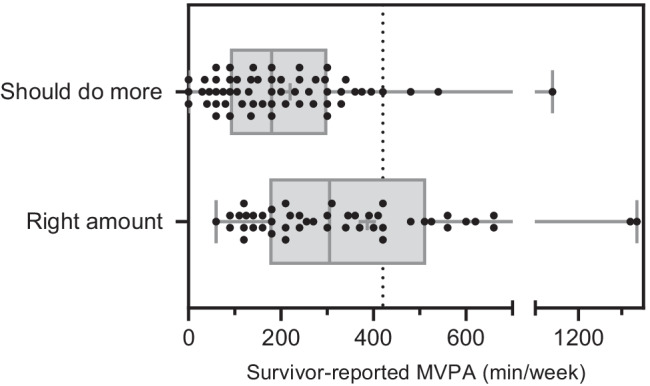
Parent perceptions regarding their child’s physical activity levels. *Abbreviations*: MVPA, moderate to vigorous physical activity. Median and range of parents’ perceptions of their child’s physical activity levels vs. survivors’ self-reported MVPA (minutes/week). The dotted line denotes the recommended guidelines for physical activity (420 min/week of MVPA) for children aged 8–17 years. Survivors who were 18 years old (*n* = 13) were adjusted for their recommended guidelines (150 min/week of MVPA). The ‘should be doing less’ group was removed due to small sample (*n* = 4)

Among parents who reported that their child ‘should do more’ physical activity, 88% of these survivors were not meeting the recommended physical activity guidelines (Fig. 1). Among parents who reported that their child did ‘do the right amount’ of physical activity, 65% of these survivors were not meeting the physical activity recommendations.

When asked whether they wanted their child to do more physical activity, most parents reported ‘definitely’ (*n* = 54, 45%) or ‘probably’ (*n* = 23, 19%). Some parents (*n* = 15, 13%) reported not wanting their child to increase their physical activity levels (Table 2).

**Fig. 2:**
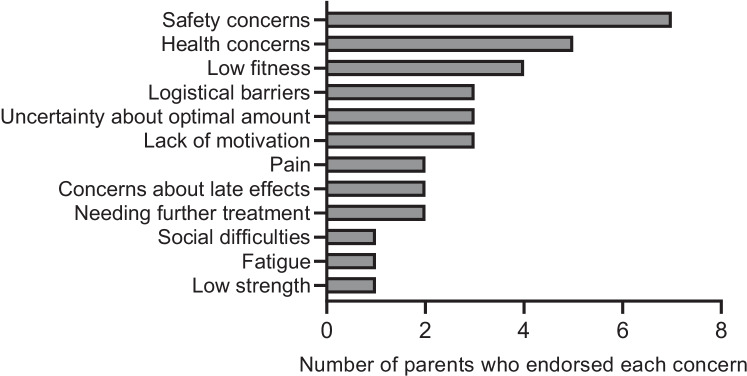
Number of parents who endorsed each concern regarding their child exercising after cancer treatment (*n* = 19). NB, parents were able to list multiple concerns when responding

Some parents (*n* = 19, 17%) reported having concerns about their child exercising after cancer treatment. Twenty-seven percent of male parents (*n* = 7/26) and 12% of female parents (*n* = 12/99) reported concerns. All parents valued the importance of their child to be physically active (100% scored more than 70/100 where 100 = extremely important), and 47% (*n* = 9) did not achieve recommended physical activity guidelines themselves (*n* = 5, 28% were sedentary [0 min/week], *n* = 4, 22% were insufficiently active [1–149 min/week]). These parents reported a mean (*SD*) of 2.8 (1.9), ranging between one to four concerns. Open-ended responses revealed that the most reported concerns about their child exercising after cancer treatment were ‘safety concerns’ (*n* = 7, 22%), ‘health concerns’ (*n* = 5, 19%), and ‘low fitness’ (*n* = 4, 13%) (Fig. 2). Examples of safety concerns included concerns about *suitable intensities of physical activity*, *contact sports*, and *resistance exercises*. Health concern examples included that their child had *musculoskeletal problems*, *low bone density*, and *cardiovascular concerns*.

### Factors associated with parent perceptions of their child’s physical activity levels

Regarding our fourth aim, we used logistic regression to examine factors (parent psychosocial outcomes and child attributes) associated with parents perceiving that their child ‘should do more’ physical activity. Parents were more likely to perceive that their child ‘should do more’ physical activity if their child was an adolescent at the time of study (*OR* = 4.952, 95% *CI* [1.801, 13.616], *P* = 0.002) and had a higher body fat percentage (*OR* = 1.082, 95% *CI* [1.029, 1.137], *P* = 0.002; Table 3). The univariable models showed that, considered alone, parents with lower scores on ‘happiness’, ‘satisfaction with life’, or ‘coping’, or higher scores of ‘stress or worry’ or ‘sadness or depression’ on the PAFAS were more likely to perceive that their child should do more physical activity (Tables 3 and 4). In the multivariable model, these associations were attenuated and were not statistically significant, and therefore we could not identify if any of the individual PAFAS aspects were driving these associations, after adjusting for the other aspects.

**Table 3:** Summary of logistic regression analysis examining associations between survivors’ clinical attributes with parents’ perceiving that their child ‘should do more’ physical activity (‘doing the right amount’ versus ‘should do more’ physical activity)

	Univariate			Multivariate		
	*OR*	95% *CI*	*P*	*OR*	95% *CI*	*P*
Survivor attributes						
Age at study^a^	2.181	1.047, 4.542	0.037	*4.952*	1.801, 13.616	*0.002*
Diagnosis group^b^	1.045	0.468, 2.330	0.915	1.305	0.431, 3.956	0.638
Months since treatment completion	0.997	0.989, 1.005	0.426	0.994	0.984, 1.004	0.229
Cancer treatment^c^						
Surgery	1.778	0.801, 3.944	0.157	2.138	0.855, 5.744	0.124
Radiation therapy	1.428	0.628, 3.247	0.395	1.359	0.393, 4.695	0.628
Bone marrow transplant	0.953	0.394, 2.305	0.953	0.589	0.147, 2.367	0.456
Body fat percentage	1.054	1.015, 1.095	0.006	1.082	1.029, 1.137	*0.002*

*CI*, confidence interval, *OR*, odds ratio. ^a^We categorised survivors’ age at study to ‘child’, defined as 8 to 12 years, and ‘adolescent’, defined as 13 to 18 years of age. ^b^Diagnosis group was coded as 0 = liquid and 1 = solid cancer group. Brain diagnosis group was removed due to low sample (*n* = 3). ^c^Chemotherapy variable for treatment was removed from this analysis as most survivors received chemotherapy

**Table 4:** Summary of logistic regression analysis examining the association between parents’ psychosocial functioning and their perception that their child ‘should do more’ physical activity (‘doing the right amount’ versus ‘should do more’ physical activity)

	Univariate			Multivariate		
	*OR*	95% *CI*	*P*	*OR*	95% *CI*	*P*
Parent psychosocial functioning (PAFAS)						
Stress or worry	1.592	1.002, 2.528	0.049	1.186	0.674, 2.087	0.553
Happy	0.362	0.181, 0.721	0.004	0.534	0.223, 1.278	0.259
Sad or depressed	1.872	1.036, 3.380	0.038	1.010	0.477, 2.178	0.979
Satisfied with life	0.466	0.264, 0.821	0.008	0.838	0.402, 1.749	0.638
Coping	0.387	0.217, 0.688	0.001	0.545	0.276, 1.075	0.080

*CI*, confidence interval; *OR*, odds ratio; *PAFAS*, Parent and Family Adjustment Scale

## Discussion

This study investigated parents’ perceptions regarding their own physical activity levels and their child’s physical activity levels. Understanding this relationship may play a key role in developing effective and safe programs to increase physical activity levels in survivors. Regarding our first aim, we identified that many survivors and parents themselves did not meet recommended physical activity guidelines. We observed a weak relationship between parents’ self-reported MVPA and survivors’ self-reported MVPA. For our second aim, we did not observe any significant relationship between parent psychosocial functioning and survivors’ self-reported MVPA. Our third aim highlighted that most parents of survivors believe that it is highly important for their child to be physically active and would like them to increase their physical activity levels. Some parents had inaccurate perceptions by perceiving that their child was sufficiently active when in fact they were not meeting the physical activity guidelines. Some parents also expressed concerns about their child exercising after cancer treatment. Regarding our fourth aim, we found that survivors who were adolescents at the time of study and their body fat percentage were associated with parents perceiving that their child should increase physical activity levels.

Most parents in this study were either sedentary or were not meeting the recommended physical activity guidelines. Engaging in regular physical activity may not be a priority for parents of survivors, as they are often the primary caregivers responsible for their child’s health during and after their child’s cancer treatment [37]. This is concerning as parents of children with disabilities or chronic conditions may experience their own negative health effects when caring for their unwell child [38, 39]. They are often profoundly affected by their child’s diagnosis and experience emotional distress or difficulties with coping due to the increased demands of managing their child’s illness and treatments, family caregiving, time pressures, and financial demands [40, 41]. Due to the high demands of time and money, providing care that aligns with the identity of being a ‘good mother’ or ‘good father’ may lead parents to prioritise their use of time and money for their child, even at the cost of their own health and wellbeing [38]. As a result, physical activity may be reduced among both parents themselves, and survivors.

In families with children not affected by cancer, previous studies have shown that parents’ physical activity levels may directly influence their child’s physical activity behaviour, otherwise known as parent modelling [11]. In line with the social cognitive theory, individuals learn behaviours by observing the behaviour of others [42]. However, our results found a weak association between parents’ and survivors’ self-reported physical activity levels. Trost and Loprinzi assessed the relationship between parents’ and their child’s physical activity levels in the general population and did not find a direct link [11]. Rather, a positive association was found with respect to parent support, suggesting that parents can still improve their child’s physical activity behaviours by directly playing with their child, in addition to watching their child play sports, providing transportation to parks and facilities, and reinforcing participation in physical activity, even if they are not physically active themselves [11]. A review of parent involvement in diet and physical activity interventions for childhood cancer survivors found that most interventions had indirect or no parental involvement [43, 44]. Studies that did involve parents in interventions for survivors showed positive outcomes, including improved fitness and increased fruit and vegetable intake [43, 44]. Thus, encouraging physical activity among the entire family unit may be a viable solution for promoting behaviour change for childhood cancer survivors.

Parents in our study reported that it was highly important for their child to be physically active and most reported a desire for their child to do more physical activity. The factors we identified using multivariable analyses for parents perceiving that their child *should* be more physically active was if their child were older and had a higher body fat percentage. Our results suggest that parents may perceive their child’s adiposity as a cue to increase physical activity levels. However, our results also identified a subgroup of survivors who may be overlooked in terms of parents’ focus on physical activity. It is possible that survivors who are sedentary but have a low body fat percentage may be missed because they may not ‘appear’ to need more physical activity, despite other peripheral benefits of physical activity besides body composition, such as cardiovascular fitness. Education about the importance of physical activity for all children, no matter their body type, may be valuable.

Some parents in our study expressed concerns about their child exercising after cancer treatment, which may reflect overprotective behaviours that mean that those parents are less likely to encourage or engage their child in physical activity. Parents’ concerns were multifactorial, most commonly regarding the safety of exercises or contact sports, and health concerns such as their child’s low bone density and cardiovascular complications. In the general population, physical activity can be used to treat and prevent chronic diseases such as cardiovascular disease and osteoporosis [45]. Preliminary evidence supports physical activity as a potential mediator of long-term effects, such as improvements in bone mineral density for survivors of childhood cancer [46]. However, common health problems in survivors may limit their ability or confidence in physical activity or sport participation, such as pulmonary disease and general performance limitations [47, 48]. Parents’ concerns for their child’s safety during exercise and their uncertainty about the optimal amount, motivation, pain, and potential late effects can be addressed through individualised consultations with an exercise professional [49]. Future research studies and interventions should consider involving exercise professionals and focus on physical activity education for parents to address common concerns and provide them with the knowledge they need to support their child to be more physically active [49].

### Strengths and limitations

This study is one of the first to explore parents’ perceptions regarding childhood cancer survivors’ physical activity levels. Some study limitations should be considered when interpreting our results. Our small sample size may affect the reliability of our multivariable analysis model. Additionally, the power of the multivariable analysis is weakened due to correlations between the various aspects of the psychosocial functioning scale. Some measures used in our questionnaire were modified to meet our research questions and target population; however, they had not been formally validated. Furthermore, we did not collect survivors’ adverse effects of treatment in this study, which may have affected parent perceptions. We collected self-reported physical activity data which may be biased and inaccurate compared to objective measurements [50]. Future studies should utilise objective assessments of physical activity where possible. We recruited mostly English-speaking, educated mothers from metropolitan areas, from a single hospital. Perceptions may differ substantially in families from different cultures or lower socio-economic backgrounds, and future studies should aim to include socially and ethnically diverse populations. Additionally, this study may have attracted families who were already interested in physical activity, thus may underrepresent those parents who place less importance on their child to be physically active. Despite these limitations, this study has important implications for understanding how parents perceive their child survivor’s physical activity levels. Understanding parents’ perceptions regarding childhood cancer survivors’ physical activity may assist in future supportive care and the development of future physical activity interventions.

## Conclusion

The findings from this study build on our understanding of how parents perceive physical activity for their child after cancer treatment. While parents reported the value of physical activity for their child, some parents expressed concerns or inaccurately perceived the amount of physical activity that their child achieves. Families may benefit from more support and guidance regarding exercise for survivors to address parents’ needs and concerns. Specifically, attention should be paid to the safety of exercises including suitability of physical activity intensities, contact sports, and resistance training. Encouraging increased physical activity levels and reduced sedentary behaviour among both parents and childhood cancer survivors may assist with improving health behaviours for the entire family unit.

## Data Availability

The data that support the findings of this study are available from the corresponding author, LH, upon reasonable request.
